# Wrist Proprioception: Amplitude or Position Coding?

**DOI:** 10.3389/fnbot.2016.00013

**Published:** 2016-10-19

**Authors:** Francesca Marini, Valentina Squeri, Pietro Morasso, Lorenzo Masia

**Affiliations:** ^1^Motor Learning and Robotic Rehabilitation Laboratory, Department of Robotics, Brain and Cognitive Sciences, Istituto Italiano di Tecnologia, Genova, Italy; ^2^Rehab Technologies, Istituto Italiano di Tecnologia, Genova, Italy; ^3^School of Mechanical and Aerospace Engineering, Nanyang Technological University, Singapore

**Keywords:** proprioception, joint position sense, human wrist, robot-aided rehabilitation, amplitude coding, final position coding

## Abstract

This work examines physiological mechanisms underlying the position sense of the wrist, namely, the codification of proprioceptive information related to pointing movements of the wrist toward kinesthetic targets. Twenty-four healthy subjects participated to a robot-aided assessment of their wrist proprioceptive acuity to investigate if the sensorimotor transformation involved in matching targets located by proprioceptive receptors relies on amplitude or positional cues. A joint position matching test was performed in order to explore such dichotomy. In this test, the wrist of a blindfolded participant is passively moved by a robotic device to a preset target position and, after a removal movement from this position, the participant has to actively replicate and match it as accurately as possible. The test involved two separate conditions: in the first, the matching movements started from the same initial location; in the second one, the initial location was randomly assigned. Target matching accuracy, precision, and bias in the two conditions were then compared. Overall results showed a consistent higher performance in the former condition than in the latter, thus supporting the hypothesis that the joint position sense is based on vectorial or amplitude coding rather than positional.

## 1 Introduction

The process by which people translate sensory impressions into a coherent and unified view of the world around them is called perception. Proprioception is the sensory stream responsible for the unconscious perception of body movements and spatial awareness (McCloskey, 1978), and it comes from nerves and organs within limbs (Riemann and Lephart, 2002b) and the inner ear (Yu Wei et al., 1986), involving the entire nervous system (Smith et al., 2009). The proprioceptive process which underlies the interpretation of body segments’ position is called joint position sense, and it plays a crucial role in all the processes related to motor control and learning (Schmidt, 1988). Neuropathies and brain injuries can massively and in some cases permanently deprive the brain of its presumed main sources of dynamogenic information from skin and muscles (Bard et al., 1995; Langhorne et al., 2009), leading to a compromised coding of the proprioceptive information (Debert et al., 2012), with negative consequences in motor control and its recovery process (Dukelow et al., 2012). In particular, it has been observed that in the absence of proprioceptive afferents, gross motor functions are preserved (Schabrun and Hillier, 2009), but given the importance of peripheral information in movement planning and control (Riemann and Lephart, 2002a), considerable motor deficits might persist (Goble et al., 2012). Therefore, a clear understanding of mechanisms giving rise to proprioceptive perception is needed but, however, the exact contribution of proprioceptive afferences, together with motor efferences, is still debated.

Reaching out an object nearby placed without looking at it is a common action of many activities of daily living. Such kind of action involves a motor plan, set up on the basis of the sensory information acquired from proprioceptors in the phase of placing (Nougier et al., 1996). Earlier literature identified two possible motor strategies for this matching movement toward a proprioceptive target (Miall and Wolpert, 1996). The first hypothesis suggests that the movement is the consequence of a prior estimation of the relative position between the limb and the target (Morasso, 1981; Wolpert et al., 1995; Miall and Wolpert, 1996), thus assuming a vectorial coding of space representation which requires information on movement amplitude (Bock and Eckmiller, 1986; Meyer et al., 1988; Ghez et al., 1995) (*amplitude coding strategy*: AC). Alternatively, several lines of evidences suggest that the central nervous system (CNS) controls limb movements by setting muscle length–tension parameter (Feldman, 1980), so that the final equilibrium point corresponds to the position of the target, whatever the initial position of the limb in space (final *position coding strategy*, PC) (Bizzi et al., 1976; Latash and Gottlieb, 1991).

Therefore, given that the AC and PC models provide divergent interpretations of the same phenomenon, there is still a lack of agreement about the importance of movement starting position in proprioceptive target perception and aiming.

In order to verify these contradictory results, we asked twenty-four participants to perform with their wrist a joint position matching task (Goble, 2010) that consisted of three phases: (1) a passive, i.e., robot driven, *criterion movement* that brought the wrist joint in a preset angular configuration that the blindfolded subjects were asked to memorize; (2) a passive *removal movement* away from the target position to a selected initial position; and (3) a *matching movement* actively performed by the subjects toward the memorized target location.

With the aim of contrasting the two alternatives formulated above (AC vs. PC), the testing paradigm included two experimental conditions, one with a constant starting position, between the *criterion* and *matching movement*, and another condition in which such position was shifted in space. Performance in the two conditions was then compared in terms of accuracy, precision, and bias. Our results indicate that the difference in starting position strongly biases the process of coding proprioceptive information of the perceived joint position, thus resulting in a lower matching performance as predicted from the amplitude-control model.

## 2 Materials and Methods

### 2.1 Participants and Experimental Setup

**Figure 1:**
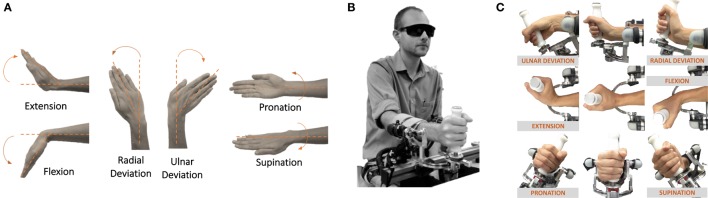
**(A)** Wrists DoFs and movements involved in the task (flexion/extension, radial/ulnar deviation, and pronation/supination). **(B)** Blindfolded participant performing the task by means of the wrist robotic device. **(C)** Kinematics of the robotic device which allows for movement along the three DoFs of the wrist.

Twenty-four subjects (mean age 29.3 ± 4.12 years, 11 females, 13 males) with no history of sensorimotor disorders were enrolled in the study. All participants were right-handed according to the Edinburgh Handedness Inventory (Oldfield, 1971). The study was approved by Ethics Committee of the regional health 61 authority, Azienda Sanitaria Locale Genovese (ASL) N.3 (Protocol number 29/08 62 approved on 10/2/2008), and all the participants signed a written informed consent. Experiments were carried out at the Motor Learning and Robotic Rehabilitation Lab of the Istituto Italiano di Tecnologia (Genoa, Italy). The experimental design involved a behavioral task where subjects were seated in front of a three-degree of freedom (DoF) wrist manipulandum (Masia et al., 2009) holding its handle with their right hand (Figure 1B). The robotic device allowed movements along the three wrist DoFs (Figures 1A,C): flexion/extension (FE: 70°), radial/ulnar deviation (RUD: 35°), and pronation/supination (PS: 80°), for almost the full range of motion (RoM) of the human wrist. It is powered by four brushless motors chosen in such a way to provide an accurate haptic rendering and compensate for the weight and inertia of the device. Angular rotations on the three axes are acquired by means of 4000 quadrature-counts/revolution incremental encoders, resulting in a resolution of 0.0075. The continuous torque ranges at the different wrist joints are 1.53 nm on FE, 1.63 nm on RUD, and 2.77 nm on PS. Subjects sat beside the robotic device with the frontal plane of their body aligned perpendicularly to the sagittal plane and the PS axis of the robotic device; relative position between the device and each participant was adjusted to have a 90° configuration for the elbow. Particular attention was dedicated to the correct alignment between the axes of the robotic system and the wrists anatomical ones; subjects forearm was firmly strapped to a mechanical support to ensure repeatability of wrist positioning and limit inter-trial variability, to avoid joints misalignment and unwanted relative movements during task execution.

The mechanical transparency of device was maximized by a control algorithm for inertia and gravity compensation in such a way to reduce force and effort during the active matching movement and to not involve other muscular activations besides those involved in the task. Furthermore, the handle of the device was carefully designed in order to allow anatomical grasp to minimize fingers stretch may leading to a high activation pattern of flexor/extensor muscles during the active phase of the matching task.

### 2.2 Task and Procedure

**Figure 2:**
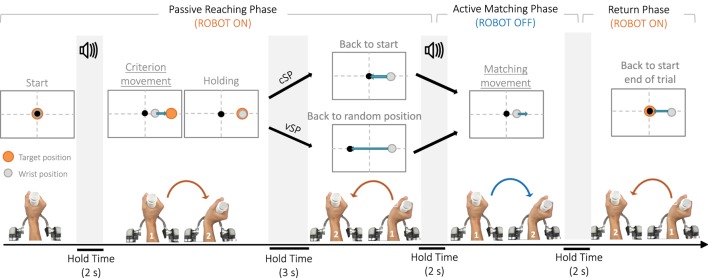
**The temporal sequence of the experimental paradigm**. An auditory cue marks the beginning of the trial, and the wrist is passively moved by the robotic device from the neutral configuration to the proprioceptive target (*criterion movement*). After a consistent holding time of 3 s, the joint is passively removed from the target (*removal movement*) and positioned or in the neutral configuration (*constant starting point condition*, cSP) or in a random one (*variable starting point condition*, vSP). Another auditory cue indicates participants to start moving and actively reproduce, the joint configuration previously experienced (matching movement). In this phase, the robot is inactive. When the end effector speed is below a 2°/s for more than 2 s, the robot moves the wrist back to the neutral position and another trial can start.

The proprioceptive test consisted in an ipsilateral joint position matching task (JPM) (Goble, 2010) in which a preset wrist configuration was passively presented to a blindfolded participant who was then asked to replicate it, as accurate as possible. Figure 2 shows the breakdown of the experimental trial: from the neutral anatomical configuration (0° of FE, 0° of RUD, and 0° of PS), the robot passively moved the wrist in a determined angular position corresponding to the proprioceptive target (*criterion movement*), maintained it there for 3 s (Fuentes and Bastian, 2010), and then removed the wrist from that position (*removal movement*) to an appropriately chosen initial position. Thereafter, the subject was required to actively reproduce the previously experienced wrist configuration, as accurately as possible (*matching movement*), and the robot was not actuated but let the subject free to move. An auditory cue (high-frequency beep) sounded to mark the beginning of the *criterion movement* and auditory cue (low-frequency beep) sounded, indicating to the subject that he could start the *matching movement*, aiming to the previously presented proprioceptive target location. The *matching movement* was considered completed when wrists speed was lower than a 2°/s threshold for more than 2 s. During the *criterion* and *removal movements*, the robot control was on and provided forces to move the wrist toward the proprioceptive target and then back along a minimum jerk trajectory, in passive way. On the contrary, the *matching movement* had to be performed actively by the subject and neither forces nor torques were provided by the haptic device, apart from those necessary for compensation of weight and inertia. The proprioceptive test involved the three DoFs separately, one at a time: the robot allowed movements only in the DoF involved in the current trial while keeping the other two in the neutral configuration.

Proprioceptive targets were located at a distance chosen as 80% of the total functional wrists RoM. In particular, these positions were: 32° (80% of 40°) for flexion and extension, 16° (80% of 20°) for radial and ulnar deviation, and 24° (80% of 30°) for pronation and supination. Subjects did not receive any feedback about their performance to eliminate the possibility they recalibrate the responses during testing, based on direct knowledge of performance. Vision was occluded for all the duration of the experimental trials.

In order to ascertain if the matching accuracy depends on information regarding solely the proprioceptive target position (*position coding strategy*, PC), or contrarily it relies also on movement performed to reach the target (*amplitude coding strategy*, AC), the designed protocol consisted of two conditions, differing in the starting positions of the *matching movements* (determined by the robot controller as final positions of the removal movements). In the PC case, the initial position was constant for all matching movements and was chosen as the neutral configuration of the wrist (cSP in Figure 2). In the AC case, on the contrary, the subjects were not repositioned in the neutral wrist configuration but in a randomly chosen variable position along the tested DoF, shifted away from the neutral one (vSP in Figure 2). Subjects were instructed to concentrate only on the *criterion movement* end location and ignore all other potential sources of information. In details, the experimenters paid particular attention to the instructions given to each participant who was requested to try to perceive and store the wrist position at the end of the *criterion movement* (passively operated by the robot) and to “repeat” it as accurate as possible. No explicit information was given to them about the existence of the two condition and movements starting location.

Proprioceptive targets were randomized across the DoFs and conditions, and each trial was repeated 12 for a total of 72 trials lasting about 30 min.

### 2.3 Outcome Measures

Wrist joint rotations were recorded from the robots incremental encoders, with a microradian resolution; the acquired signals were post-processed by a third-order Savitzky–Golay low-pass filter (cut-off frequency of 10 Hz) and to angular wrist joint displacements from the kinematics of the robot. To estimate the proprioceptive acuity of the wrist position sense and characterize the overall performance, three indicators were evaluated: the *matching error* (Schmidt, 1988), the *error variability* (Dukelow et al., 2010), and the *error bias* (Schmidt, 1988).

The *matching error* measures the performance accuracy, and it is computed as the absolute deviation between the proprioceptive target position and the wrist configuration at the end of the matching movement:

(1) $$\mathrm{Matching}\;\mathrm{Error}=\frac{\sum_{i=1:N}\;|\theta_{i}-\theta_{T}|}{N}$$

where *θ_i_* is wrists final position of the i-trial, *θ_T_* is the target position, averaged across the N (=12) repetitions of the same target (same DoF and condition).

The *variability* is evaluated, for each DoF, as the SD across the N (=12) trials of wrists position at the end of the *matching movement*, thus providing information about subjects’ performance consistency (or precision):

(2) $$\mathrm{Variability}=\mathrm{StD}\left(\theta_{i}-\theta_{T}\right)$$

The *error bias* indicating bias in subjects’ performance is determined as the algebraic distance between the ideal proprioceptive target and the final wrist position at the end of the *reaching movement*. It indicates the subjects’ tendency of undershooting (negative *error bias*) and overshooting (positive *error bias*) the target. It is evaluated as follows:

(3) $$\mathrm{Error}\;\mathrm{Bias}=\frac{\sum_{i=1:N}\;\left(\theta_{i}-\theta_{T}\right)}{N}$$

## 3 Results

### 3.1 Accuracy: The *Matching Error*

**Figure 3:**
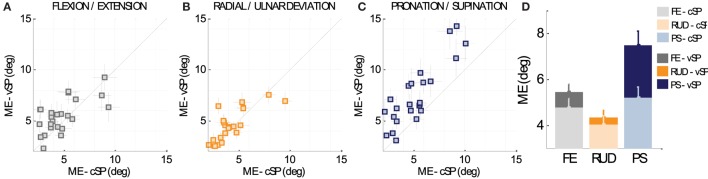
**(A–C)** Comparison between *matching errors* in the cSP and vSP condition for FE, RUD, and PS, respectively, for the 24 subjects. Data points which fall below the 45° line (equality line) indicate larger *matching errors* in the cSP condition, whereas data points which fall above the equality line indicate lowest performance in PC condition, when starting points of criterion and matching movements are different (vSP condition). Data points that fall directly on the equality line indicate no preference between cSP and vSP condition. **(D)** Overall difference for the three DoFs between *matching errors* in the cSP and vSP condition.

These plots compare the *matching error* in the two conditions (cSP and vSP, respectively), for the 24 subjects: for the sake of clarity, data points which fall below the 45° line (equality line) indicate larger *matching errors* in the cSP condition, whereas data points which fall above the equality line indicate lowest performance in vSP condition, and data points that fall directly on the equality line indicate no preference between the cSP and vSP conditions. It is clear from Figure 3D that smaller *matching errors* were found in the cSP condition for all of the three DoFs: FE, RUD, and PS. Such a predominant trend of lower accuracy in the vSP condition can be seen for most of the subjects, as shown in Figures 3A–C for FE, RUD, and PS, respectively. A 2-way ANOVA test (conditions × DoFs) confirmed these results, revealing a main effect of condition (*F*_1,144_ = 10.694, *p* = 0.00135) showing a significance difference between *matching errors* in AC (4.65° ± 0.30°) and PC condition (5.76° ± 0.30°).

### 3.2 Matching Precision: Variability

**Figure 4:**
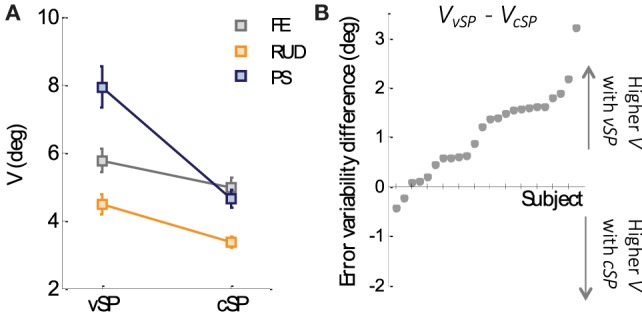
**(A)** *Variability* cSP and vSP condition for the three DoFs. **(B)** Difference, for each subject, between the *variability* of the vSP and the cSP condition. Subjects’ data resulting positive indicate a higher *variability* in the vSP condition, whereas data resulting lower than zero indicate higher *variability* in vSP condition.

Figure 4A reports population values of the *variability* V or the *matching error* in the two conditions (cSP and vSP, respectively). This analysis of *variability* indicates that movements in the cSP condition were more precise (*variability* = 4.32° ± 0.18°) than those performed in the vSP condition (*variability* = 6.07° ± 0.35°). Such difference in *variability* resulted highly significant from a 2-way ANOVA test (conditions × DoFs) revealing a main effect of condition (*F*_1,144_ = 36.785, *p* < 0, 001) independently from the three DoFs that resulted to have the same trend of higher precision (lower *variability*) in the cSP condition. Figure 4B plots the difference of V in the two conditions for all the subjects, ordered from the minimums to the maximum difference: it appears that almost all the subjects (22 out of 24) revealed a greater *variability* in the vSP condition, thus indicating a consistent tendency of being more precise when the starting point of matching and criterion movement is the same (cSP).

### 3.3 Tendency in Target Overshooting/Undershooting: The *Error Bias*

In the figure is reported the error bias of each subject for the three DoFs (light gray, light orange, and light blue for FE, RUD, and PS, respectively), the average of all the participants for the three DoFs with the bigger dots (gray for FE, orange for RUD, and blue for PS), and three ellipses which highlight, for each DoF, the area in which fall the 50% of the data of the 24 participants.

**Figure 5:**
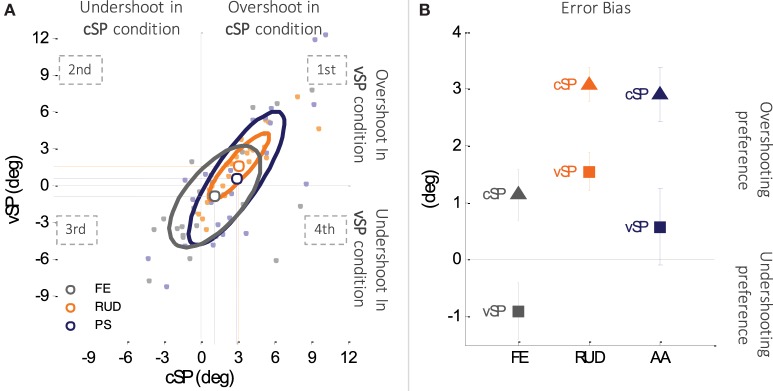
**(A)** Subjects tendency of target undershooting (negative *error bias*) or overshooting (positive *error bias*) in the two matching conditions (cSP and vSP). x-axis reports errors made in the cSP condition and the y-axis those made in the vSP condition. Subjects are represented with small dots (light gray, light orange, and light blue for FE, RUD, and PS, respectively), and the three ellipses represent the distribution of the *error bias* in the two conditions for the three DoFs separately. Data points falling in the first or third quadrant of the Cartesian plane indicate the same tendency target overshooting or undershooting, respectively, in both cSP and vSP condition. Conversely, data points in the second quadrant indicate predominance of target undershooting in cSP but not in the vSP condition and, vice versa, data points in the fourth quadrant indicate predominance of target undershooting in vSP but not in the cSP condition. **(B)** Overall changes in targets under and overshooting between cSP and vSP condition.

Figure 5A is a scatter plot of the *error bias* in the two conditions (cSP and vSP, respectively) for the three DoFs (FE, RUD, and PS) and all the subjects. The corresponding *error bias* plane is naturally divided into four quadrants: in the first quadrant, the subjects overshoot the target position in both conditions; in the second quadrant, there is an overshoot in the vSP condition and an undershoot in the cSP; in the third quadrant, there is an undershoot in both conditions; and in the fourth quadrant, there is an undershoot in the vSP condition and an overshoot in the cSP. It appears that the great majority of data points, representing the *error bias* of each subject for the particular DoF, falls in the first and third quadrants, i.e., the subjects have the same tendency (to under or overshoot) in both conditions. Figure 5B plots population values of the *error bias*, separately for the three DoFs. Overall, the three DoFs presented a strong tendency to overshoot proprioceptive targets if the starting points of criterion and matching movements were unchanged (cSP), vice versa in case of variable starting point (vSP) subjects were still biased toward targets overshooting, but with lower consistency. Contrarily, for flexion/extension, for targets in the vSP condition a predominant undershoot was observed. A 2-way ANOVA test (conditions × DoFs) revealed a high significant main effect of condition (*F*_1,144_ = 9.6632, *p* = 0.00227), which was independent of the DoF since no significant interaction was found between DoF and conditions. The overall *error bias* in the vSP condition was 0.44° ± 0.65°, while in the cSP condition, it increased up to 2.36° ± 0.51°, confirming that overshooting is more consistent in cSP condition, while in vSP condition, subjects have a less predominant strategy.

## 4 Discussion

Apart from purely physiological aspects, the motivation of this study is primarily related to the fact that a large body of knowledge has been accumulated in recent years as regards the crucial role of proprioception in promoting or hindering motor learning (Ostry et al., 2010; Wong, 2012) and influencing neuromotor rehabilitation. In the case of stroke, for example, in addition to motor dysfunctions, a majority of patients are affected by kinesthetic impairments, and there is solid prognostic evidence that an intact position sense strongly correlates with the likelihood of motor recovery of the hemiplegic arm (Kusoffsky et al., 1982; Smith et al., 1983; Rand et al., 1999). In spite of this, traditional robot and virtual reality training techniques focus on the recovery of motor functions and not on the assessment of proprioceptive deficits, although some preliminary exceptions can be quoted (Taub and Berman, 1963; Casadio et al., 2009; De Santis et al., 2015). Hemiparetic patients tend to over-rely on visual feedback in order to surrogate the missing or impaired proprioceptive feedback, and thus, a reasonable goal in robotic rehabilitation can be formulated as follows: how can we enhance the relative importance of proprioceptive information, with respect to vision, during a robotic assisted training protocol? This question motivated a preliminary empirical study (Casadio et al., 2009) based on an experimental design that allows to carry out trials of robot assistance with the traditional vision-based feedback and trials performed without vision, with full immersion in a purely proprioceptive virtual world.

However, in order to go beyond the empirical stage based on some assessment of the degree of sensorimotor and functional improvements in neuromotor impaired subjects, determined by techniques of proprioceptive training, it is necessary to understand in a better and deeper manner the physiological mechanisms of proprioceptive perception. This paper is therefore a step in this direction, although further studies are necessary. We focused indeed on the coding mechanism of proprioceptive information, based on proprioceptive memory but in the absence of visual feedback.

To understand the mechanism underlying the process of matching proprioceptive targets, we designed an experiment to highlight the differences between position and amplitude cues. Indeed, up-to-date, the type of information which is encoded by the motor system is still under debate. Specifically, in the present study, we sought to investigate if the accuracy in proprioceptive target matching depends only on the target final position or whereas the amplitude of the criterion movement is a relevant factor. In order to address this issue, we compared performance in two conditions: constant starting point, cSP (*criterion* and *matching movements* starting from the same position) and variable starting point, vSP (criterion movement starting from the neutral configuration and *matching movement* starting from a random position).

Lower performance in the second condition (vSP) indicates that subjects do not rely only on the final position of the proprioceptive target, and information about movement amplitude is fundamental for a better perception (*amplitude coding* strategy). Conversely, an unchanged performance between vSP and cSP condition indicates that sensory signals acquired in the final location are sufficient for an accurate perception (* final position coding* strategy) and no other information is needed.

Overall, we observed that proprioceptive abilities in target matching depend significantly on the mode of target presentation, and our results support the hypothesis that signals in the brain are coded in terms of amplitude rather than position. The present findings show indeed that when subjects experience a shift in the starting position, a systematic pattern of increasing errors emerged, indicating a tendency to use the *amplitude coding* strategy rather than the *position coding* one. Several interpretations can lay down this phenomenon, ranging from psychophysics to motor control.

From a psychophysics perspective, a widely recognized approach has conceptualized the movement as a process involving three entities: starting position, distance moved, and terminal location (Walsh et al., 1979). If we accept this interpretation, it is licit to consider the proprioceptive information as something beginning to arise as the movement starts and keeps on consolidating until the movement itself reaches the end location. During this time, a complex set of signals is conveyed from the proprioceptors to the central nervous system where they give rise to the perception of target position. Such signals consist in the mechanical interactions existing between them and the surrounding tissues (muscle, tendon, ligament, bones, or skin) but also in position, velocity, and force information that are mixed in a history-dependent manner for the entire movement duration. In line with this idea, our results showed a consistent loss in accuracy and precision when the starting point of the matching movement is shifted, and therefore its amplitude is different, supporting the hypothesis of an amplitude coding interference on position coding.

Besides this psychophysics interpretation, a neuroscientific explanation can be provided to the decrement in performance emerged when the starting position of the matching movement is randomly shifted in space, and it deals with the source of noise arising in this condition to the motor system. The motor system is the one responsible for computing the sensorimotor transformation, between sensory inputs and motor commands, but the sensory signals it constantly deals with are generally corrupted by noise. Noise permeates every level of the nervous system, from sensory to motor responses generation, posing a fundamental problem for information processing (Faisal et al., 2008). Recent studies have begun to reveal computational principles by which the central nervous system tries to minimize the sensory uncertainty (Bays and Wolpert, 2007) and movement *variability* arising from this internal noise (Bays and Wolpert, 2007). It is certain that the most inaccurate and variable (noisy) are the sensory signals that form the input, the higher is the uncertainty in the estimation of the state (Bard et al., 1995). The shift in the starting point of the criterion movement can be considered as a noisy information for three main reasons: first, because of the random nature of the shift magnitude from trial to trial; second, because it is an unexpected event the unaware subject has to deal with; and finally, the source of noise in the sensory signal may be introduced by the lack of stable space landmarks, such as the movement starting position, despite to the normal condition in which both the matching and the criterion movement start from the neutral wrist configuration.

Furthermore, from a biomechanical perspective, we should consider that the inability to properly calibrate the motor response given the sensory input might be associated with the loss of calibration of the central control signals in terms of spatial coordinates (Flanagan et al., 1993). Indeed, in the cSP condition, the starting positions of both the criterion and matching movements correspond with the neutral wrist configuration (0° FE, 0° RUD, 0° PS), which is likely the one adopted as the center of the reference frame built in the neural processing that involves the transformation from the sensory information to the motor command. It could be reasonable to identify the difficulty in properly coding the target location with variable starting point in the mismatch between the starting position and the reference frame built and adopted by each subject.

Besides being due to changes in actual kinesthetic signals arising from criterion and matching movements, the systematic pattern of accuracy decrement in end location reproduction could also have a cognitive component. Several authors suggested indeed that the interference between distance and location seems to occur not only at a sensory or perceptual level but also at a more abstract or cognitive level of information processing (Kerr, 1978; Faisal et al., 2008) with a consistent influence on the strategy adopted for the sensory encoding and consequent movement planning (Walsh et al., 1979; Imanaka and Abernethy, 1992).

Changes in *error bias* uphold the hypothesis of an amplitude-coding strategy preference: when the starting point of the matching moment is shifted away from the one of the criterion movement (vSP), a longer movement is requested to accurately match the end location. If subject could isolate the information about the final position (preference for position rather than amplitude coding), the strong tendency of target overshooting observed in the cSP condition should persist. On the contrary, observed results highlighted how when the requested movement is longer (starting point shifted away), the tendency for overshooting decreases, and in the case of flexion/extension, targets are over and undershot with the same frequency, meaning that subject is not able to dissociate the information about movement extent.

## 5 Conclusion

Our findings reduce significantly the plausibility of a pure final position coding hypothesis, at least in experimental situation in which the role of proprioceptive memory is emphasized; in contrast, the results are more compatible with a general qualitative role played by proprioceptive afferents in informing the CNS about the evolving state of the peripheral system as well as of the outcome of motor commands (Rothwell et al., 1982; Sanes et al., 1985; Gandevia and Burke, 1992). A natural evolution of this study is to investigate issues of proprioceptive–visual calibration and how they can affect the proprioceptive coding problem.

## Author Contributions

FM: substantial contributions to the conception or design of the work; or the acquisition, analysis, or interpretation of data for the work; and drafting the work or revising it critically for important intellectual content. VS: contributions to the conception or design of the work; and interpretation of data for the work. PM: important contribution in revising the work critically for important intellectual content. LM: contribution in revising the work critically and final approval of the version to be published. All the authors: agreement to be accountable for all aspects of the work in ensuring that questions related to the accuracy or integrity of any part of the work are appropriately investigated and resolved.

## Conflict of Interest Statement

The authors declare that the research was conducted in the absence of any commercial or financial relationships that could be construed as a potential conflict of interest.

## References

[B1] Bard C. Fleury M. Teasdale N. Paillard J. Nougier V. (1995). Contribution of proprioception for calibrating and updating the motor space. Can. J. Physiol. Pharmacol. 73, 246–254. 10.1139/y95-035 7621363

[B2] Bays P. M. Wolpert D. M. (2007). Computational principles of sensorimotor control that minimize uncertainty and variability. J. Physiol. 578, 387–396. 10.1113/jphysiol.2006.120121 17008369 PMC2075158

[B3] Bizzi E. Polit A. Morasso P. (1976). Mechanisms underlying achievement of final head position. J. Neurophysiol. 39, 435–444. 815518 10.1152/jn.1976.39.2.435

[B4] Bock O. Eckmiller R. (1986). Goal-directed arm movements in absence of visual guidance: evidence for amplitude rather than position control. Exp. Brain Res. 62, 451–458. 10.1007/BF00236023 3720877

[B5] Casadio M. Morasso P. Sanguineti V. Giannoni P. (2009). Minimally assistive robot training for proprioception enhancement. Exp. Brain Res. 194, 219–231. 10.1007/s00221-008-1680-6 19139867

[B6] De Santis D. Zenzeri J. Casadio M. Masia L. Riva A. Morasso P. (2015). Robot-assisted training of the kinesthetic sense: enhancing proprioception after stroke. Front. Hum. Neurosci. 8:1037. 10.3389/fnhum.2014.01037 25601833 PMC4283673

[B7] Debert C. T. Herter T. M. Scott S. H. Dukelow S. (2012). Robotic assessment of sensorimotor deficits after traumatic brain injury. J. Neurol. Phys. Ther. 36, 58–67. 10.1097/NPT.0b013e318254bd4f 22592061

[B8] Dukelow S. P. Herter T. M. Bagg S. D. Scott S. H. (2012). The independence of deficits in position sense and visually guided reaching following stroke. J. Neuroeng. Rehabil. 9, 72. 10.1186/1743-0003-9-72 23035968 PMC3543214

[B9] Dukelow S. P. Herter T. M. Moore K. D. Demers M. J. Glasgow J. I. Bagg S. D. (2010). Quantitative assessment of limb position sense following stroke. Neurorehabil. Neural Repair 24, 178–187. 10.1177/1545968309345267 19794134

[B10] Faisal A. A. Selen L. P. J. Wolpert D. M. (2008). Noise in the nervous system. Nat. Rev. Neurosci. 9, 292–303. 10.1038/nrn2258 18319728 PMC2631351

[B11] Feldman A. (1980). Superposition of motor programs – I. Rhythmic forearm movements in man. Neuroscience 5, 81–90. 10.1016/0306-4522(80)90073-1 7366845

[B12] Flanagan J. R. Ostry D. J. Feldman A. G. (1993). Control of trajectory modifications in target-directed reaching. J. Mot. Behav. 25, 140–152. 10.1080/00222895.1993.9942045 12581985

[B13] Fuentes C. T. Bastian A. J. (2010). Where is your arm? Variations in proprioception across space and tasks. J. Neurophysiol. 103, 164–171. 10.1152/jn.00494.2009 19864441 PMC4116392

[B14] Gandevia S. C. Burke D. (1992). Does the nervous system depend on kinesthetic information to control natural limb movements? Behav. Brain Sci. 15, 614–632. 10.1017/S0140525X0007254X

[B15] Ghez C. Gordon J. Ghilardi M. F. (1995). Impairments of reaching movements in patients without proprioception. II. Effects of visual information on accuracy. J. Neurophysiol. 73, 361–372. 7714578 10.1152/jn.1995.73.1.361

[B16] Goble D. J. (2010). Proprioceptive acuity assessment via joint position matching: from basic science to general practice. Phys. Ther. 90, 1176–1184. 10.2522/ptj.20090399 20522675

[B17] Goble D. J. Mousigian M. A. Brown S. H. (2012). Compromised encoding of proprioceptively determined joint angles in older adults: the role of working memory and attentional load. Exp. Brain Res. 216, 35–40. 10.1007/s00221-011-2904-8 22006273

[B18] Imanaka K. Abernethy B. (1992). Cognitive strategies and short-term memory for movement distance and location. Q. J. Exp. Psychol. A 45, 669–700. 10.1080/14640749208401338 1484977

[B19] Kerr B. (1978). The effect of invalid task parameters on short-term motor memory. J. Mot. Behav. 10, 261–273. 10.1080/00222895.1978.10735160 15186988

[B20] Kusoffsky A. Wadell I. Nilsson B. Y. (1982). The relationship between sensory impairment and motor recovery in patients with hemiplegia. Scand. J. Rehabil. Med. 14, 27–32. 7063817

[B21] Langhorne P. Coupar F. Pollock A. (2009). Motor recovery after stroke: a systematic review. Lancet Neurol. 8, 741–754. 10.1016/S1474-4422(09)70150-4 19608100

[B22] Latash M. L. Gottlieb G. L. (1991). An equilibrium-point model for fast, single-joint movement: II. Similarity of single-joint isometric and isotonic descending commands. J. Mot. Behav. 23, 179–191. 10.1080/00222895.1991.10118361 14766515

[B23] Masia L. Casadio M. Sandini G. Morasso P. (2009). Eye-hand coordination during dynamic visuomotor rotations. PLoS ONE 4:e7004. 10.1371/journal.pone.0007004 19753120 PMC2737429

[B24] McCloskey D. I. (1978). Kinesthetic sensibility. Physiol. Rev. 58, 763–820. 360251 10.1152/physrev.1978.58.4.763

[B25] Meyer D. E. Abrams R. A. Kornblum S. Wright C. E. Smith J. E. K. (1988). Optimality in human motor performance: ideal control of rapid aimed movements. Psychol. Rev. 95, 340–370. 10.1037/0033-295X.95.3.340 3406245

[B26] Miall R. Wolpert D. (1996). Forward models for physiological motor control. Neural Netw. 9, 1265–1279. 10.1016/S0893-6080(96)00035-4 12662535

[B27] Morasso P. (1981). Spatial control of arm movements. Exp. Brain Res. 42, 223–227. 10.1007/BF00236911 7262217

[B28] Nougier V. Bard C. Fleury M. Teasdale N. Cole J. Forget R. (1996). Control of single-joint movements in deafferented patients: evidence for amplitude coding rather than position control. Exp. Brain Res. 109, 473–482. 10.1007/BF00229632 8817278

[B29] Oldfield R. C. (1971). The assessment and analysis of handedness: the Edinburgh inventory. Neuropsychologia 9, 97–113. 10.1016/0028-3932(71)90067-4 5146491

[B30] Ostry D. J. Darainy M. Mattar A. A. G. Wong J. Gribble P. L. (2010). Somatosensory plasticity and motor learning. J. Neurosci. 30, 5384–5393. 10.1523/JNEUROSCI.4571-09.2010 20392960 PMC2858322

[B31] Rand D. Tamar Weiss P. L. Gottlieb D. (1999). Does proprioceptive loss influence recovery of the upper extremity after stroke? Neurorehabil. Neural Repair 13, 15–21. 10.1177/154596839901300104

[B32] Riemann B. L. Lephart S. M. (2002a). The sensorimotor system, part II: the role of proprioception in motor control and functional joint stability. J. Athl. Train. 37, 80–84. 16558671 PMC164312

[B33] Riemann B. L. Lephart S. M. (2002b). The sensorimotor system, part I: the physiologic basis of functional joint stability. J. Athl. Train. 37, 71–79. 16558670 PMC164311

[B34] Rothwell J. C. Traub M. M. Day B. L. Obeso J. A. Thomas P. K. Marsden C. D. (1982). Manual motor performance in a deafferented man. Brain 105, 515–542. 10.1093/brain/105.3.515 6286035

[B35] Sanes J. N. Mauritz K. H. Dalakas M. C. Evarts E. V. (1985). Motor control in humans with large-fiber sensory neuropathy. Hum. Neurobiol. 4, 101–114. 2993208

[B36] Schabrun S. M. Hillier S. (2009). Evidence for the retraining of sensation after stroke: a systematic review. Clin. Rehabil. 23, 27–39. 10.1177/0269215508098897 19114435

[B37] Schmidt R. A. (1988). Motor Control and Learning: A Behavioral Emphasis, 2nd Edn USA: Human Kinetics Publishers.

[B38] Smith D. L. Akhtar A. J. Garraway W. M. (1983). Proprioception and spatial neglect after stroke. Age Ageing 12, 63–69. 10.1093/ageing/12.1.63 6846094

[B39] Smith J. L. Crawford M. Proske U. Taylor J. L. Gandevia S. C. (2009). Signals of motor command bias joint position sense in the presence of feedback from proprioceptors. J. Appl. Physiol. 106, 950–958. 10.1152/japplphysiol.91365.2008 19118155

[B40] Taub E. Berman A. J. (1963). Avoidance conditioning in the absence of relevant proprioceptive and exteroceptive feed back. J. Comp. Physiol. Psychol. 56, 1012–1016. 10.1037/h0048315 14100937

[B41] Walsh W. D. Russell D. G. Imanaka K. James B. (1979). Memory for constrained and preselected movement location and distance. J. Mot. Behav. 11, 201–214. 10.1080/00222895.1979.10735188 23962288

[B42] Wolpert D. M. Ghahramani Z. Jordan M. I. (1995). An internal model for sensorimotor integration. Science 269, 1880–1882. 10.1126/science.7569931 7569931

[B43] Wong J. (2012). On Sensorimotor Function and the Relationship Between Proprioception and Motor Learning. Electronic Thesis and Dissertation Repository.

[B44] Yu Wei J. Simon J. Randic M. Burgess P. (1986). Joint angle signaling by muscle spindle receptors. Brain Res. 370, 108–118. 10.1016/0006-8993(86)91110-8 2939921

